# Multi-Disciplinary Care Planning of Ovarian Cancer in Older Patients: General Statement—A Position Paper from SOFOG-GINECO-FRANCOGYN-SFPO

**DOI:** 10.3390/cancers14051295

**Published:** 2022-03-02

**Authors:** Leila Bengrine, Naoual Bakrin, Frédérique Rousseau, Vincent Lavoué, Claire Falandry

**Affiliations:** 1 SOFOG, GINECO, Medical Oncology Department, Centre Georges-Francois Leclerc, 21000 Dijon, France; lbengrine@cgfl.fr; 2 GINECO, Hospices Civils de Lyon, Digestive Surgery Department, Hôpital Lyon-Sud, CEDEX, 69495 Pierre-Bénite, France; naoual.bakrin@chu-lyon.fr; 3 SOFOG, GINECO, Paoli Calmettes Institute, 13009 Marseille, France; rousseauf@ipc.unicancer.fr; 4 FRANCOGYN, Gynecology Department, CHU de Rennes, Hôpital Sud, 35000 Rennes, France; vincent.lavoue@chu-rennes.fr; 5 UMR S1085, IRSET-INSERM, Rennes University, 35000 Rennes, France; 6 SOFOG, GINECO, Hospices Civils de Lyon, Geriatrics Department, Hôpital Lyon Sud, 69230 Saint Genis-Laval, France; 7 CarMeN Laboratory, INSERM U.1060, Université Lyon1, INRA U. 1397, INSA Lyon, Hospices Civils Lyon, Bâtiment CENS-ELI 2D, Hôpital Lyon Sud Secteur 2, 69310 Pierre Bénite, France; 8 UCOGIR—Auvergne-Rhône-Alpes Ouest–Guyane, Charles Mérieux Medical School, ONCOAGE FHU, 69310 Pierre Bénite, France

**Keywords:** ovarian cancer, older patient, oncogeriatrics, geriatric assessment, vulnerability, care plan, strategy

## Abstract

**Simple Summary:**

This position paper aims to provide practitioners a proposal for multidisciplinary care planning for older patients with ovarian cancer from the time of suspected diagnosis. The first-line treatment of advanced ovarian cancer involves several interdependent sequences: cytoreductive surgery, (neo)adjuvant chemotherapy and maintenance targeted treatments. In older patients, care planning must be adapted to their geriatric parameters and consider the geriatric impact of each treatment sequence to allow treatment completion. Care planning should be centered on patient motivation and imply multidisciplinarity. Each step of treatment plan should be reconsidered in light of a geriatric assessment and follow-up. Studies are needed to prospectively evaluate the impact of geriatric vulnerability parameters at each step of the treatment agenda and the impact of geriatric interventions on patient outcomes.

**Abstract:**

In this position paper the Société Francophone d’OncoGériatrie (SOFOG; French-speaking oncogeriatric society), the Société Française de Pharmacie Oncologique (SFPO, French society for oncology pharmacy), the Groupe d’Investigateurs Nationaux pour l’Étude des Cancers de l’Ovaire et du sein (GINECO, National Investigators’ Group for Studies in Ovarian and Breast Cancer) and the Groupe Français de chirurgie Oncologique et Gynécologique (FRANCOGYN) propose a multi-disciplinary care planning of ovarian cancer in older patients. The treatment pathway is based on four successive decisional nodes (diagnosis, resectability assessment, operability assessment, adjuvant, and maintenance treatment decision) implying multidisciplinarity and adaptation of the treatment plan according to the patient’s geriatric covariates and her motivation towards treatment. Specific attention must be paid to geriatric intervention, supportive care and pharmaceutical conciliation. Studies are needed to prospectively evaluate the impact of geriatric vulnerability parameters at each step of the treatment agenda and the impact of geriatric interventions on patient outcomes.

## 1. Introduction

Management of advanced ovarian cancer has been progressively standardized over recent decades to the association of an extensive cytoreductive surgery and adjuvant chemotherapy; this allowed overall survival rates to improve, and the median survival now exceeds 40 months [1]. Cancer control is also further prolonged in patients receiving bevacizumab and Poly (ADP-ribose) polymerase inhibitors (PARPi) as maintenance therapies [2]. Nevertheless, the reported overall survival of older patients, in population-based studies and even in randomized trials, remains poor due to the presence of comorbidities, polypharmacy and frailty in this population, as these factors impede optimal management [3,4,5,6]. This may be explained by a priori concerns; for example, age is frequently associated with poorer application of recommendations [3] but also a posteriori such as excessive toxicities, leading to dose limitations, treatment discontinuation and even debates about the treatment paradigms in the oldest old [7]. However, optimal management is defined according to data collected in younger populations since older patients are seldomly included in pivotal trials [8] and when included represent a highly selective population, differing from “true” older patients [9,10,11]. Data for the treatment of older patients are derived from retrospective studies of selected populations [12], subgroup analyses of pivotal randomized trials [13,14], real-life unselected population-based studies [5] and specific clinical trials conducted in older patients, but these seldomly integrate assessment of geriatric covariates. These are often small and non-comparative phase II studies, and there is heterogeneity in the geriatric covariates explored. In this context, results are often not comparable, and guidelines may be difficult to elaborate. Nevertheless, this question stays of major interest, since the median age of ovarian cancer at diagnosis reached 68 years in France in 2019 [15].

The international community has identified geriatric oncology as a priority for many tumors, and the Gynecological Cancer InterGroup (GCIG) identified during its fourth consensus conference the need to develop research involving older patients with the following objectives: (i) reduce selection bias in pivotal trials; (ii) assess better and prospectively, using stratification methods, geriatric covariates in such trials and (iii) conduct specific trials devoted to older patients that may not be included in randomized studies due to different characteristics or poorer prognosis [16]. More recently the French national cancer institute (Institut national du cancer, INCa) coordinated national recommendations on ovarian cancer and dedicated a specific chapter to older patients [17]. In parallel, the geriatric oncology community aims to share the objectives of geriatricians that are to avoid under-treatment, over-treatment and bad practices; an additional challenge is to provide older patients with access to therapeutic innovation.

This position paper aims, in this context of a relatively low level of evidence, to provide practitioners a proposal for multidisciplinary care planning for older patients with ovarian cancer from the time of suspected diagnosis. It summarizes the proposals of a working group composed of members of two learned societies and two investigator groups: the Société Francophone d’OncoGériatrie (SOFOG; French-speaking oncogeriatric society), the Société Française de Pharmacie Oncologique (SFPO, French society for oncology pharmacy), the Groupe d’Investigateurs Nationaux pour l’Étude des Cancers de l’Ovaire et du sein (GINECO, National Investigators’ Group for Studies in Ovarian and Breast Cancer), and the Groupe Français de chirurgie Oncologique et Gynécologique (FRANCOGYN, French research group for oncologic gynecologic surgery). The patient’s care course is hereafter conceptualized as the succession of several decisional nodes in which the classical risk/benefit ratio is enriched by the adjunct of geriatric assessment and, when data are available, the way such geriatric assessment may modify patients’ treatment schedule.

## 2. General Considerations for Anticipating the Treatment Care Planning Agenda

Ovarian cancer in older patients is frequently perceived as a dramatic change in the physiological trajectory of the patient, a perfect illustration of frailty and the so-called “geriatric cascade” or “domino effect” [18]. Due to cancer-related covariables (for example, frequent diagnosis at a later stage of tumors that are histologically more aggressive [19,20]) or to patient-related covariables, older patients suffering from ovarian cancer present frequently at diagnosis a rapid functional, nutritional and psychological deconditioning [21]. The challenges of cancer treatment therefore reside in a personalized, anticipated and adaptive treatment plan that should integrate two opposing constraints: Provide to each patient the best specific oncological treatment based on evidence-based medicine without precipitation of geriatric deconditioning or functional loss. Other points to consider are, at each decision step, the opinion of the patient and her family on cancer treatment, their motivation towards specific treatment and geriatric rehabilitation, as well as the shared objectives of the treatment plan, with a particular attention paid to quality of life and functional preservation more than quantity of life, and supportive care [22].

### What Are the General Principles of Such Treatment Care Planning?

Some concepts may be highlighted to provide a general framework for the principles of care.

First, complete surgery provides the same overall survival benefit to older patients as it does to their younger counterparts [23]. However, due to a higher risk of perioperative morbidity and mortality [5] this benefit becomes clear 16 months after surgery; moreover, surgery is less frequently complete in older patients [3]. In addition, surgery induces not only a high risk of perioperative morbidity and mortality but also geriatric deconditioning [24] and impairs the dose intensity of subsequent chemotherapy [7,25]. Consequently, decisions on surgery should be made as often as possible based on preliminary laparoscopic assessment, and starting cytoreductive surgery that may not be completed should be discouraged [26].

Second, some categories of patients do not benefit from upfront surgery, namely patients older than 75 years of age with stage IV ovarian cancer or with stage III ovarian cancer and evolving comorbidity [27]. For these patients, upfront chemotherapy offers a greater incremental cost-effectiveness ratio (ICER) [28]. In addition, patients with a geriatric vulnerability score (GVS) ≥ 3 (i.e., three or more impaired geriatric parameters among the following: altered activities of daily living (ADL) or instrumental ADL (IADL), hypoalbuminemia, altered hospital anxiety and depression scale (HADS) or lymphopenia) are a significant risk of premature death [21] and should probably not be offered upfront surgery.

Third, when upfront surgery is performed, the risk is high that adjuvant chemotherapy is delivered later [25], using monotherapy [7], and with a reduced dose intensity [25].

Fourth, neoadjuvant chemotherapy provides an opportunity to decrease the complexity of cytoreductive surgery and perioperative complications [29], implement a geriatric plan that will address specifically the geriatric covariates identified during the initial geriatric assessment and optimize patients’ status before surgery (when interval surgery is expected to be proposed).

**Figure 1:**
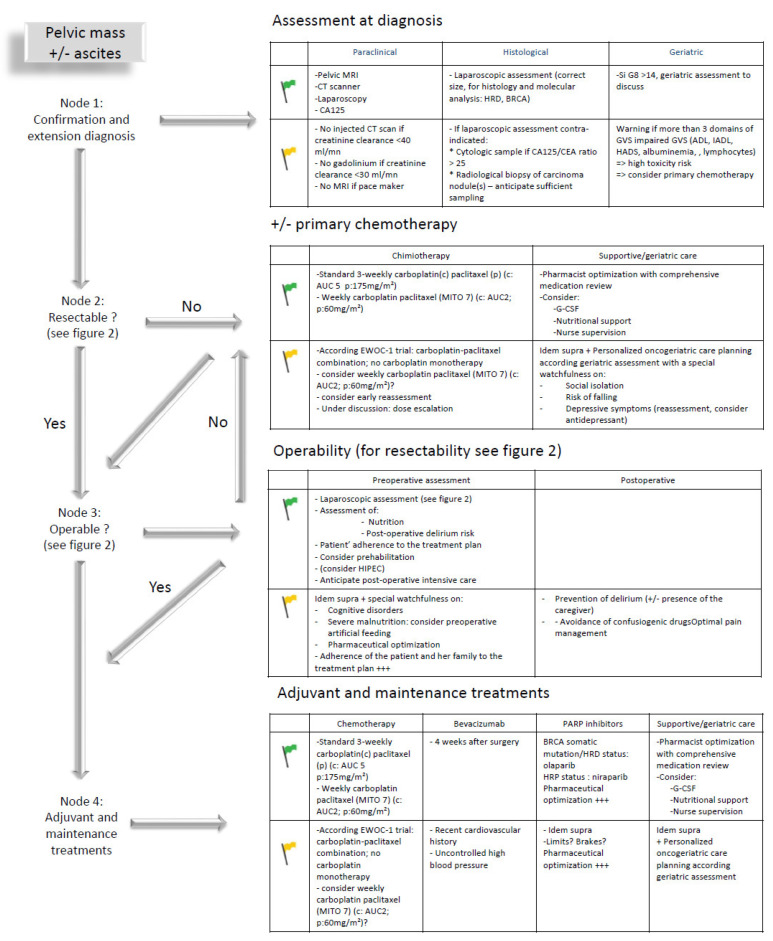
Care planning in older patients with advanced ovarian cancer: steps to be considered from diagnosis to treatment in case of geriatric green (fit patient) or yellow (vulnerable patient) flags; ADL: activities of daily living; AUC: area under the curve; CA-125: carcinoma antigen 125; CEA: carcinoembryonic antigen; EWOC-1: elderly women with ovarian cancer trial 1; IADL: instrumental ADL; GVS: geriatric vulnerability score; HADS: Hospital Anxiety and Depression Scale; HRD: homologous recombination deficient; HRP: homologous recombination proficient; MITO-7: Multicenter Italian Trial in Ovarian Cancer study 7; ?: to be discussed; +++: important or even necessary.

Based on these broad outlines, each treatment decision should consider tumor-specific and patient-specific characteristics to coordinate an individualized treatment plan coordinating the different ”blocks” of treatment that are developed below in the following order: diagnosis, chemotherapy, surgery, adjuvant and maintenance treatments including targeted therapies (Figure 1). Considering the lack of data available to distinguish different outcomes of older patients receiving chemotherapy with neoadjuvant, adjuvant or palliative (without any surgical plan) intent, all data are gathered under the section entitled chemotherapy.

In addition, and as a first step to enter such an individualized care plan, the benefit of performing a geriatric assessment from the time of diagnosis and of implementing a geriatric care plan coordinated with the oncological plan is presented.

## 3. Diagnosis

### 3.1. Histological Diagnosis

Epithelial ovarian cancer classically requires the association of a radiological pelvic mass and evocative histological sampling compatible with a gynecological origin [30]. Such sampling may come from a laparoscopic assessment, providing both a histological and extension diagnosis or, when the risk/benefit ratio is unsatisfactory for general anesthesia, from image-guided sampling. When histological sampling is considered high risk, an evocative cytology associated with a CA125/CEA ratio greater than 25 is accepted as an alternative [31]. However, image-guided techniques may not be able to provide sufficiently large tissue samples for treatment individualization. The risk is high that such non-invasive sampling methods are favored for older populations leading to a putative reduced access to therapeutic innovation (PARPi, clinical trial participation, etc.). Consequently, classical histological sampling should always be encouraged, and cytology considered as an exception (Figure 1, node 1).

### 3.2. Operability and Resectability

Upfront cytoreductive surgery followed by six courses of platinum-based chemotherapy is the standard treatment of patients with resectable stage III advanced ovarian cancer. However, the high proportion of advanced disease in older women and its general and geriatric consequences often prevent upfront surgery. In such cases neoadjuvant chemotherapy may be considered as an alternative treatment option [26,29]. The decision for neoadjuvant chemotherapy integrates both the resectability of the tumor and operability of the patient. Resectability refers to the ability of the surgeon to perform a complete cytoreduction (evaluated using the Essen or Leuven criteria [32]), whereas operability addresses the issue of whether a surgical plan is safe for an individual patient; the latter is based in particular on geriatric vulnerabilities. Thus, nutritional status, comorbidities and general condition assessment are needed to assess operability, as it has been shown that elevated Charlson comorbidity index and hypoalbuminemia are independent prognostic factors associated with severe complications after upfront surgery [33]. Some histological subtypes, such as mucinous, clear cell and low-grade serous carcinoma, have low response rates to chemotherapy; such patients should be proposed for maximal effort of complete surgical cytoreduction given the poor benefit of chemotherapy in these cases. In other cases, neoadjuvant chemotherapy must be considered when the tumor is not resectable and/or the patient is not operable; the latter must be reassessed after three courses of chemotherapy to propose interval surgery.

## 4. Chemotherapy

### 4.1. Defining the Regimen with the Best Efficacy to Tolerability Profile

Considering both the substantial extent of cancer at diagnosis and geriatric factors of vulnerability, primary chemotherapy is frequently considered as a reasonable therapeutic option, either with a neo-adjuvant or a palliative intent, when surgery is definitively rejected. Geriatric cofactors may also impact chemotherapy tolerance and challenge the predefined standards of treatment. From the beginning of the 1990s, the GINECO led several prospective studies of the Elderly Women with Ovarian cancer Trials (EWOT) program to define the geriatric covariates associated with treatment tolerance and patients’ outcomes. A first study, EWOT-1, evaluated the treatment completion rate of a combination of cyclophosphamide and carboplatin; the prognostic factors for lower overall survival were depression, a high level of comedication and cancer stage; toxicity rates were higher when patients presented depression or instrumental ADL impairment [34]. This was followed by EWOT-2, which evaluated the treatment completion rate of the standard 3-weekly paclitaxel 175 mg/m^2^ and carboplatin area under the curve (AUC) 5 mg/mL·min combination. Again, depression—according the investigator and evaluated using HADS, was a prognostic factor for overall survival [35]. When considering a pooled analysis of EWOT-1 and EWOT-2 results, factors associated with overall survival were depression, lymphopenia, stage IV cancer and the use of paclitaxel. The third study, EWOT-3, evaluated the treatment completion rate of a carboplatin monotherapy, with a special attention to depression assessment and to the impact of a standardized geriatric assessment on treatment completion and toxicity rates. The study led to the development of a geriatric vulnerability score (GVS) that includes five vulnerability covariates: ADL score < 6/6; IADL score < 25/27, albuminemia < 35 g/L, lymphopenia < 1 G/L and HADS score > 14/42; patients are considered as vulnerable if they have at least three of these parameters (GVS ≥ 3) [21]. These were followed by the Multicentre Italian Trial in Ovarian Cancer (MITO)-5 study that demonstrated that weekly administration of paclitaxel and carboplatin in older patients (paclitaxel 60 mg/m^2^ and carboplatin AUC 2 mg/mL·min on D1, D8 and D15 every 4 weeks) was feasible as it was associated with a low frequency of unacceptable toxicity (11.5%) [36].

Based on the data from these four reports, the randomized phase II EWOC-1 study was designed to evaluate the treatment completion rates of a standard 3-weekly paclitaxel carboplatin regimen, a weekly paclitaxel carboplatin regimen as in the MITO-5 study and a carboplatin monotherapy in patients with a GVS ≥ 3. Single-agent carboplatin was less active with significantly worse survival outcomes suggesting that even vulnerable patients should receive a combined treatment [37].

### 4.2. Supportive Care Must Be Associated with Chemotherapy

Chemotherapy needs to be associated with current standards of supportive care developed in the younger populations and completed with geriatric interventions proposed during the comprehensive geriatric assessment, especially nutritional care, fall prevention and management of social isolation [38].

Supportive care should prevent serious adverse events of chemotherapy. Hematologic toxicity will be prevented by prophylactic hematopoietic growth factors and digestive toxicity by systematic anti emetic treatment. Daily nursing supervision should be set up as soon as the patient is discharged from hospital. This supervision will allow close monitoring of chemotherapy’s tolerance, blood pressure and temperature [39]. Pharmacists can also be implicated in this follow-up, notably in case of treatment delivery, to promote the patient’s adherence and pharmaceutical conciliation.

## 5. Surgery

Curative surgery in ovarian carcinoma aims to achieve a complete clearance of the abdominal cavity [23]. The extent of residual disease is a strong prognostic factor [5,23], and in cases of low chemosensitivity maximal surgical effort can even restore prognosis [40]. However, this complex surgery includes digestive tract, urological and peritoneal procedures and is challenging in older women with comorbid conditions and high risk of post-operative complications [41,42]. Incomplete surgery is more frequent [43], partially due to surgeons modifying their cytoreductive approach, which seems to lead to decreased bowel resections [3]. Post-operative morbidity may delay the onset of adjuvant chemotherapy [44], decrease its dose intensity [7] and increase chemotherapy-related toxicity [25,45], leading some authors to question the treatment paradigm that includes surgery for the oldest patients [7].

Some studies have attempted to define predictive factors of excess morbidity in women undergoing cytoreduction. For instance, Wright et al. identified age, comorbidity and the number of procedures performed as the strongest predictors of medical complications that ranged from 10.2% in patients aged <50 years of age who underwent 0 radical procedures to 33.3% for patients aged ≥80 years with ≥2 procedures [46]. According to Gerestein et al. post-operative morbidity can be predicted by age, performance status, extent of surgery and operative time [47]. Taken together, age and surgical complexity appear as robust predictors of post-operative morbidity. It is also of note that Gerestein et al. had previously reported in a systematic analysis on post-operative mortality that the overall mean was 2.8% and that it was 3.7% for primary surgery [48], illustrating the decreased complexity and morbidity of interval compared to primary surgery [29]; the authors also highlighted the importance of the technical platform and the expertise of the surgical team [48], which may explain the wide range of mortality between studies notably in the oldest patients (≥80 years) that ranged from 5.4% at 30 days for Diaz-Montes et al. [20] to 11.7% for Cloven et al. [49]. Moore et al. reported 13% deaths prior to hospital discharge and 30% within 60 days of surgery [7]. In parallel to these aspects, there is also growing evidence that aging is heterogeneous and that chronological age alone may not be sufficient to predict the ability to withstand operational stress [41]; the worse prognosis for older patients with ovarian cancer probably encompasses both the lack of optimal treatment in fit elderly patients and inadequate surgery in frail ones, with dramatic consequences for subsequent chemotherapy and reduced survival [44].

Taken together, in older patients the treatment decision must therefore consider the risk/benefit ratio of cytoreductive surgery, considering an excess in short-term (perioperative) morbidity and an equivalent benefit over the long term of complete cytoreductive surgery [5,23]. The multidisciplinary decision for surgery should include surgical complexity and tumor load, expertise of the surgical team and the technical platform, comprehensive geriatric assessment and patient’s motivation towards surgery (importantly including prehabilitation). The purpose of preoperative assessment is to identify patients with an elevated risk of poor outcomes. Current guidelines for preoperative assessment in general [50], but also specifically in older patients [51,52], focus on defining single end-organ functional deficits (i.e., cardiac complications), notably illustrated by the American Heart Association guidelines for perioperative cardiovascular evaluation [50]. Although single organ evaluation cannot be ignored in the older patient population, recognition of preoperative geriatric markers related to frailty may provide additional insight in predicting poor outcomes [53], thus guiding preoperative decision-making. The motivation of the patient towards surgery has to be specifically explored, including a discussion on the risk of postoperative functional decline, loss of independence and skilled care burden, as highlighted by the Best Practices Guidelines on optimal perioperative management of the geriatric patient from the American College of Surgeons NSQIP and the American Geriatrics Society [54]. Pre-surgical assessment should also highlight the personal involvement of the patient in her own care through nutritional and functional prehabilitation as well as the application of enhanced recovery after surgery. The European Society of Gynaecologic Oncology defined in 2016 surgical team quality indicators for advanced ovarian cancer surgery [55]. Among these indicators the first refers to achievement of complete cytoreduction (minimal required target of 50% and an optimal target of 65%), the second to caseload in the center (minimal required target of 20 cases and an optimal target of 100 cases annually) and the third to training and experience of the surgeon (target of more of 90% of ovarian cancer cytoreductions performed by a trained surgeon specifically dedicated to gynecological cancer management). These recommendations should even be reinforced in the elderly: On the one hand, the expertise of the team has a major impact on the control of postoperative morbidity and the completeness of cytoreduction; on the other hand, elderly patients are more frequently managed by non-oncologists (such as general surgeons and obstetricians/gynecologists [56]) on an emergency basis for cancer complications (occlusion, perforation, infection) and are less likely to undergo surgery in a university hospital [20].

An additional level of complexity in the treatment discussion has come from the OVHIPEC study that found that hyperthermic intraperitoneal chemotherapy (HIPEC) with thiosulfate for nephroprotection improved survival in first-line initially non-resectable ovarian cancer; surgery-related morbidity and mortality at 30 days were comparable in the two arms, there was no significant difference in the rate or delay to return to intended adjuvant chemotherapy and there was a positive impact of HIPEC on recurrence-free and overall survival (over a median follow-up of 4.7 years) [57]. Patients included were aged up to 76 years; exploratory analyses on survival found a trend towards a decreased benefit of receiving HIPEC in those aged ≥65 years (HR = 0.82, 95% CI (0.40–1.68)) compared to younger patients (HR = 0.60, 95% CI (0.34–1.05)) [57]. In a meta-analysis on cytoreductive surgery and HIPEC regardless of the indication which considered different cut-offs (65, 70, 75 years) to analyze the effect of age on patients’ outcomes and toxicities, the 70 year threshold appeared to be clinically relevant; the 30-day postoperative grade 3 morbidity and the 90-day postoperative mortality were significantly higher in patients ≥70 years, although this was not associated with a longer hospital stay [58]. In a recent single-institution prospective analysis of HIPEC in patients with gynecologic cancers, Chambers et al. found that chronological age (<65 vs. ≥65 years) did not predict post-operative complications, progression-free survival or overall survival but that patients aged ≥70 years had a reduced progression-free survival following HIPEC compared to patients 65–69 years without any impact on overall survival [59]. However, the same group later reported that a composite index of 11 medical comorbidities (the modified frailty index, mFI) significantly predicted post-operative complications in multivariate analysis, with a more than 9-fold risk of grade ≥2 complications in those with mFI ≥ 2 vs. 0–1 (OR 9.4, 95% CI [3.3; 26.4], *p* < 0.001), whereas age did not [60].

**Figure 2:**
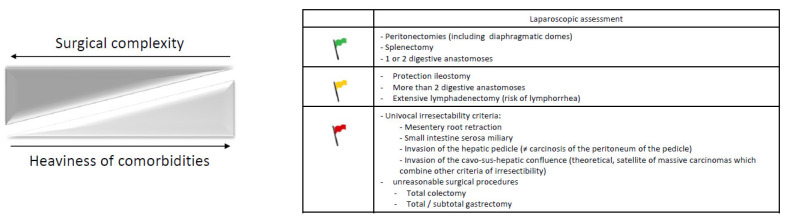
Decision factors to be considered before cytoreductive surgery in older patients with advanced ovarian cancer: the (surgical) green, yellow and red flags.

Cytoreductive surgery with or without HIPEC should, therefore, be decided during a multidisciplinary meeting based on patient motivation, based on a laparoscopic assessment of surgical complexity, the preoperative geriatric and anesthetic assessment and the experience of the team who will perform the surgery. The surgical decisional node (Figure 2) is of major importance considering its expected consequences for short- and long-term morbidity and mortality. Available data on advanced ovarian cancer cytoreductive surgery in older patients are more extensively developed in a future position paper on the surgical agenda of older patients with ovarian cancer endorsed by the SOFOG-GINECO-FRANCOGYN-SFPO.

## 6. Adjuvant Treatments: Considering the Place of Targeted Therapies in First Line

In the setting of cytoreductive surgery with no residue (CC0) surgery, adjuvant treatment and maintenance have been revolutionized by targeted agents. The first was bevacizumab, a VEGF inhibitor that has shown benefit in terms of progression-free survival but not in overall survival in stage-IIIc and IV ovarian cancer [61,62] (Table 1). In the international single-arm ROSiA study on the safety of extending bevacizumab-containing therapy up to 24 months, a post hoc analysis found that grade ≥3 adverse events such as hypertension and thromboembolic events were significantly more frequent in patients aged ≥70 years, but progression-free survival (secondary endpoint) was similar according to this age stratification [63]. The Tolerability of Bevacizumab in Elderly Ovarian Cancer Patients (TURBO) study, which evaluated real-life bevacizumab administration in maintenance, found that factors, such as higher creatinine serum levels, eGFR and number of comorbidities, and not age, were associated with bevacizumab-related grade ¾ toxicity [64]. In this context, bevacizumab should be considered for older patients in the postoperative context taking into account geriatric evaluation considering the absence of overall survival benefit and the risk of adverse events.

A greater improvement came from the availability of PARPi, a novel class anticancer agent targeting homologous recombination [65]. Two different PARPi have been approved for the treatment of first line advanced ovarian cancer, olaparib and niraparib. The study of Olaparib Maintenance Monotherapy in Patients with BRCA Mutated Ovarian Cancer Following First Line Platinum Based Chemotherapy (SOLO1) and Niraparib in Patients with Newly Diagnosed Advanced Ovarian Cancer (PRIMA) studies found a considerable benefit of olaparib and niraparib on tumor control and overall survival, respectively, in BRCA-mutated tumors [66,67]; in BRCA wild-type tumors, a significant benefit was also demonstrated with niraparib in the PRIMA study compared to a carboplatin-paclitaxel control arm. In 2019, the PAOLA study found a benefit of the olaparib and bevacizumab combined maintenance over bevacizumab only in *BRCA*-mutated and homologous recombination-deficient (HRD) tumors; there was no benefit in the homologous recombination-proficient tumors [2]. This novel therapeutic option leads to a greater individualization of patient treatment, but at the same time increases the complexity of treatment decisions as more information is required to be collected; each patient—even at older ages—should be proposed somatic/germinal analysis of the tumor, seeking *BRCA* 1 and 2 mutation, but also HRD/P score. An oncogenetic consultation is also recommended for patients over 70 with a family history.

The Young International Society of Geriatric Oncology (SIOG) Interest Group reviewed in 2019 the data available in clinical trials on age-specific tolerance of olaparib (eight phase ½ trials) and niraparib; if no difference in toxicity was shown between <65 and ≥65 [68], the authors highlighted that only a very small proportion of the patients were >75, none were >85 and almost all had an ECOG performance status of 0 or 1 [69]. A subsequent subgroup analysis of the Maintenance Study with Niraparib Versus Placebo in Patients With Platinum Sensitive Ovarian Cancer (NOVA) trial in patients aged ≥70 years with recurrent ovarian cancer found that older patients benefited from niraparib with comparable frequency, severity of adverse events (34.4%, 13.1% and 16.4% grade ≥3 thrombopenia, anemia and neutropenia events, respectively), and dose reductions [14] (Table 2).

Although chronologic age itself does not seem to significantly increase toxicities in response to PARPi (no preventive dose adjustment is necessary for older patients), specific attention must be paid to the high prevalence of chronic fatigue with a major impact in an older population [69] and to the risks of increased exposure to the treatment in case of comorbidities or polypharmacy due to drug–drug interactions and renal or hepatic impairment [70]. Differences in toxicity profiles of the drugs could be related to their pharmacokinetics. As with many oral targeted therapies, association has been shown between drug exposure and increasing probability of experiencing adverse effects. The exposure to olaparib is indeed increased in case of moderate renal impairment (dose adjustment) [71]; the area-under-the curve of niraparib depends on age, creatinine clearance, weight, albuminemia [72] and increases in case of moderate hepatic impairment (leading to a recommended starting dose of 200 mg) [73]. Niraparib tolerance was shown to be optimized with an individualized starting dose of 200 instead of 300 mg in case of persistent thrombopenia after chemotherapy <150,000/µL and/or bodyweight <77 kg whatever the patient’s age [74,75]. Considering their metabolism, olaparib and niraparib have substantial differences: olaparib is mainly metabolized by cytochrome (CYP) 3A4/5 enzymes; is a substrate of P-glycoprotein (P-gp); induces in vitro CYP1A2, 2B6 and 3A4 and inhibits in vitro CYP3A, P-gp, BCRP, OATP1B1, OCT1, OCT2, OAT3, MATE1 and MATE2K. Niraparib is mainly metabolized by carboxylesterases; it is a substrate of P-gp and BCRP and inhibits MATE1/2 and OCT1 [70]. In a multidisciplinary context, pharmaceutical consultation may be implemented to secure and optimize PARPi treatment and associated supportive care, especially in older population. The high risk of drug–drug interactions should offer the opportunity, in the older population, to a pharmaceutical optimization step with a complete comprehensive medication review including non-prescription and complementary (herbal) medications [76]. Patient education represents a significant proportion of treatment success [77], and a clear treatment plan needs to be established in the relationship between the patient and her caregivers with explanation of side effects and prevention and adaptation of the treatment plan to the vulnerabilities identified during the geriatric assessment. After prolonged exposure, PARPi-treated patients were shown to have an increased risk of myelodysplastic syndromes (OR 2·63 [95% CI 1.13–6.14], *p* = 0.026) [78]. Future phase IV studies will be of interest to evaluate whether, as expected, this risk is increased at older ages. Patients should be clearly informed, and the hematological biological monitoring should be performed carefully.

Studies are currently ongoing with immunotherapy alone or in combination with bevacizumab and PARPi. The results, if positive, could again lead to change the strategy in the first line setting.

## 7. Conclusions

Multi-disciplinarity, anticipation and patient motivation are the key rules during care planning of ovarian cancer in older patients. Each step of the treatment plan should be reconsidered in the light of a geriatric assessment and follow-up. Specific studies remain sparse and should be encouraged in the future to evaluate the impact of geriatric vulnerability parameters at each step of the treatment agenda and the impact of geriatric interventions on patients’ outcomes.

## Figures and Tables

**Table 1:** Randomized studies investigating bevacizumab associated with chemotherapy and maintenance in first-line advanced ovarian cancer—older patient subgroup analyses.

Study	Regimen	Population, *n*	Older Patients, *n*	PFS Population, Months	Median OS, Months
ICON 7r	CP vs. CP + Bev → Bev maintenance	1528	Not reported	17.4 vs. 19.8	44.6 vs. 45.5
GOG 218	CP + placebo vs. CP + Bev vs. CP + Bev → Bev maintenance	1873	>70 years: *n* = 210	10.3 vs. 11.2 vs. 14.1 HR 0.717 *p* < 0.001	39.3 vs. 38.7 vs. 39.7

Bev: bevacizumab; CP: carboplatin paclitaxel; OS: overall survival; PFS: progression free survival.

**Table 2:** Randomized studies on PARPi maintenance in first-line advanced ovarian cancer—older patient subgroup analyses.

Study	Population, *n*	Older Patients, *n*	Hazard Ratio (HR) for Disease Progression or Death	Grade 3–4 Adverse Events in Older Patients If Known
SOLO1	391	≥65: 68	HR 0.45 95% CI (0.22–0.92)	Anemia general population 22% Neutropenia 9%
PRIMA	733	≥65: 219	HR 0.53 95% CI (0.38–0.74)	Anemia 31% Thrombopenia 28.7%
NOVA	311 + 61	≥70: 61	BRCAm: HR 0.09 (95% CI: 0.01–0.73) PFS: not reached vs. 3.7 months BRCAw: HR 0.35 95% CI (0.18–0.71) PFS: 11.3 months vs. 3.8 months	Thrombopenia 34.4% Neutropenia 16.4% Fatigue 8.2%

BRCAm: BRCA mutated; BRCAw: BRCA wild type; PFS: progression free survival.

## Data Availability

Not applicable.
